# Changes in Maternal Heart Rate Variability in Response to the Administration of Routine Obstetric Medication in Hospitalized Patients: Study Protocol for a Cohort Study (MAMA-Heart Study)

**DOI:** 10.3390/clinpract11010004

**Published:** 2021-01-21

**Authors:** Maretha Bester, Suzanne Moors, Rohan Joshi, Thomas J. Nichting, M. Beatrijs van der Hout-van der Jagt, S. Guid Oei, Massimo Mischi, Rik Vullings, Judith O. E. H. van Laar

**Affiliations:** 1 Department of Electrical Engineering, Eindhoven University of Technology, 5612 AP Eindhoven, The Netherlands; suzanne.moors@mmc.nl (S.M.); thomas.nichting@mmc.nl (T.J.N.); M.B.v.d.Hout@tue.nl (M.B.v.d.H.-v.d.J.); guidoei@outlook.com (S.G.O.); m.mischi@tue.nl (M.M.); R.Vullings@tue.nl (R.V.); judith.van.laar@mmc.nl (J.O.E.H.v.L.); 2 Department of Family Care Solutions, Philips Research, 5656 AE Eindhoven, The Netherlands; rohan.joshi@philips.com; 3 Department of Obstetrics and Gynecology, Máxima MC, 5504 DB Veldhoven, The Netherlands; 4 Department of Biomedical Engineering, Eindhoven University of Technology, 5612 AP Eindhoven, The Netherlands

**Keywords:** gynecology, pregnancy complications, medical device, biomedical engineering, heart rate variability, photoplethysmography, corticosteroids, medications, hypertensive disorders of pregnancy, preterm birth

## Abstract

Pregnancy is a period of continuous change in the maternal cardiovascular system, partly mediated by the autonomic nervous system. Insufficient autonomic adaptation to increasing gestation is associated with pregnancy complications, such as hypertensive disorders of pregnancy and preterm birth (both major causes of perinatal morbidity and mortality). Consequently, maternal heart rate variability (mHRV), which is a proxy measure for autonomic activity, is increasingly assessed in these cohorts to investigate the pathophysiology of their complications. A better pathophysiological understanding could facilitate the early detection of these complications, which remains challenging. However, such studies (typically performed in pregnancies leading to hospitalization) have generated conflicting findings. A probable reason for these conflicting findings is that these study cohorts were likely administered routine obstetric medications during the study period of which the effects on mHRV are largely unknown. Subsequently, we design a longitudinal, observational study to quantifying the effect of these medications—particularly corticosteroids, which are known to affect fetal HRV—on mHRV to improve the interpretation of past and future studies. We will enroll 61 women admitted to a tertiary obstetric unit with an indication to receive corticosteroids antenatally. Participants’ mHRV will be continuously acquired throughout their hospitalization with wrist-worn photoplethysmography to facilitate a within-patient comparison of the effect of corticosteroids on mHRV.

## 1. Introduction

Pregnancy is a period of continuous anatomical and physiological change in both mother and fetus [1]. During this period, most maternal physiological systems undergo considerable adaptation to support the growing fetus. Some of the most prominent changes needed to sustain the increasing metabolic demands of the maternal-fetal dyad occur in the maternal cardiovascular system [1,2,3]. 

These maternal cardiovascular adaptations involve, amongst others, changes in blood pressure and heart rate (HR) [1]. The main mechanisms mediating these changes are related to the endocrine and the autonomic nervous systems (ANS) [2,4]. However, in some cases, the ANS does not sufficiently adapt to support the increasing demands of pregnancy—a scenario which is associated with various pregnancy complications [5]. Two prominent examples are hypertensive disorders of pregnancy (HDP) and preterm birth (PTB), both of which are leading causes of worldwide perinatal and maternal morbidity and mortality [6,7,8,9]. 

Alleviating the burden of HDP and PTB (i.e., birth before 37 weeks of gestation) remains an important challenge in perinatology, in large part because the early detection of these complications is challenging. The early detection of these conditions is important and actionable since effective risk-mitigating interventions do exist [10,11,12]. Although their exact etiologies remain uncertain, studies indicate that both complications are associated with dysfunctional autonomic regulation [5,13,14,15,16]. A prominent theory is that this autonomic dysfunction results in insufficient placental development in early pregnancy, which in turn results in the development of such complications [17,18,19]. Therefore, assessing ANS activity during pregnancy is relevant as it can allow for the tracking of developing pathophysiologies, potentially enabling early detection. 

Since changes in HR are closely modulated by the ANS, studying HR and, in particular, its variability offers a window into changes in autonomic activity [17,20,21,22]. Consequently, maternal heart rate variability (mHRV) has been increasingly studied to assess autonomic dysfunction in complicated pregnancies [5,17,19,20,23,24,25]. However, such studies, which are typically performed in pregnancies leading to hospitalization, have generated conflicting findings [17,19]. 

The onset of HDP and PTB is typically sudden, resulting in swift hospitalization to obstetric care units (OCUs), where patients frequently receive routine obstetric medications. A probable reason for these conflicting findings is that, during the study period, these study cohorts were likely administered obstetric medications that potentially confounded measures of mHRV.

Typically, soon after admission to an OCU, corticosteroids and tocolytics are administered to the patient. Corticosteroids are aimed at maturing the fetal respiratory system in the case of premature delivery [26], while tocolytics attenuate maternal contractions to reduce the risk of preterm delivery [11]. Additionally, magnesium sulfate (MgSO₄) and antihypertensive drugs may be administered as needed. These medications offer maternal and fetal neuroprotection in cases of HDP and PTB, respectively [11,12,27]. 

Consequently, studies assessing mHRV in hospitalized cohorts with complications, such as HDP and threatened PTB, likely also capture the potential confounding effects of obstetric medications. While some researchers avoid this problem by only conducting short measurements before the administration of medications [5,20], several studies do not discuss the administration of corticosteroids or tocolytics, even though their study populations would typically have received these [5,11,12,23,24,27,28,29]. Others note the potential confounding effects of these medications as an unavoidable part of their study design [30,31]. In fact, some even urge investigation into the effects of obstetric medications on mHRV [19,24]. Quantifying these changes would not only enhance our understanding of how obstetric medications affect maternal physiology but may also improve the interpretation of past and future studies. 

To our knowledge, only two studies have investigated the changes in mHRV in response to the administration of routinely used obstetric medications. Koenen et al. found no changes to the diurnal rhythm of mHRV in response to betamethasone administration (*n* = 16), although it should be noted that only short and long-term variability (STV and LTV) were assessed [32]. Additionally, Weissman et al. found that a tocolytic drug (atosiban) did not affect mHRV in hospitalized patients [33]. Even though little is known on how mHRV is affected, the effect of obstetric medications on fetal HRV (fHRV) has been more widely investigated. Similar to Weissman’s findings on mHRV, administering tocolytic drugs did not significantly alter fHRV [34]. However, corticosteroids are known to significantly affect fHRV [35,36,37,38]. Therefore, we investigate whether mHRV changes in response to administering corticosteroids. 

Investigating the effect of routine obstetric medications, such as corticosteroids and tocolytic drugs on mHRV, should contribute to understanding the impact of these medications. Subsequently, in this paper, we describe a study to investigate changes in mHRV in response to administering routinely administered obstetric medications in a cohort of patients hospitalized with pregnancy complications.

## 2. Materials and Methods

### 2.1. Aim of the Study 

This study aims to investigate the effect of routinely administered obstetric medications on mHRV in patients hospitalized due to pregnancy complications. 

### 2.2. Clinical Setting

This longitudinal, observational cohort study will be conducted at the OCU of Máxima Medical Center (Máxima MC), Veldhoven, The Netherlands. The study cohort will comprise patients admitted to the OCU between 23 5/7 and 33 6/7 weeks of gestation with an indication to receive corticosteroids antenatally. Since Máxima MC is a tertiary obstetric referral center, the majority of the study cohort will comprise high-risk patients transferred to Máxima MC from neighboring secondary care hospitals.

### 2.3. Clinical Data Acquisition

**Figure 1:**
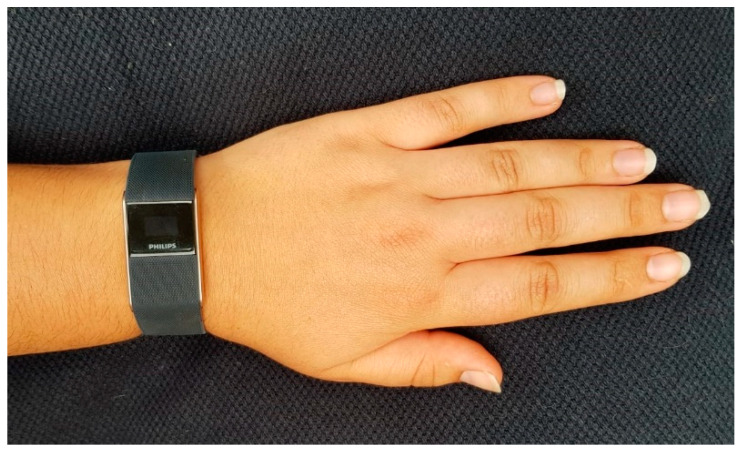
The Philips Data Logger (worn on the author’s hand). This device will be employed in this study to acquire PPG and accelerometer data. The device does not display this PPG and accelerometer data, it only displays the time.

Longitudinal PPG measurements will be continuously acquired with the Philips Data Logger (PDL, Philips Research, Eindhoven, The Netherlands, where two of the authors are affiliated). The PDL—shown in Figure 1—is a non-invasive wrist-worn device (CE-marked) that acquires PPG data (sampled at 32 Hz) through optical sensing that measure changes in blood volume. Previous studies have used and validated a predecessor of this device to collect PPG measurements in free-living conditions [39,40,41]. 

PPG measurements capture the time intervals between pulses resulting from subsequent heartbeats, serving as a measure of HR, from which HRV can be calculated [42]. Furthermore, the PDL also records movement data using a tri-axial accelerometer (range: ±8 G, sampled at 32 Hz), which can aid in filtering out motion artifacts. The PDL offloads acquired data to a mobile phone via Bluetooth. Data are not displayed on either the PDL or the mobile phone, ensuring that acquired data cannot influence clinical decision making. 

In addition to PPG measurements, the study utilizes patient data routinely collected in electronic patient files. These data—detailed in the Study Parameter section—include maternal–fetal health parameters and routine measurements. 

### 2.4. Routinely Administered Medications in Obstetric Care Settings

Owing to their clinical state, the patient cohort participating in this study will be administered one or more obstetric medication as part of their standard clinical care. All medications administered during this study are part of standard care and not influenced by study participation. 

When pregnancy complications are diagnosed before 34 weeks of gestation, patients receive corticosteroids (specifically betamethasone) [11]. Owing to its frequent use and its effects on fHRV, our study design focuses on this medication. A course of betamethasone (Celestone Chrondose^®^, Schering AG, Berlin, Germany) consists of two 11.4 mg injections administered intramuscularly, each consisting of 50% betamethasone phosphate for quick uptake (≈1 h) and 50% betamethasone acetate for slow release to facilitate sustained exposure [43,44,45]. Although the pharmacokinetics of betamethasone in the maternal system is not fully known, the maximum effect and terminal half-life of betamethasone (i.e., time until the drug concentration in plasma reduces by 50%) are believed to lie within 0.5–3 h and within 6–12 h after administration, respectively [45,46,47,48]. Betamethasone’s biological half-life—which relates to its effect on the hypothalamus-pituitary-adrenal axis—is 36–59 h [49,50], and is cleared from the maternal system within 48 h [46]. 

Patients will typically receive other obstetric medications in addition to corticosteroids; this is unavoidable in these cohorts [11,12,27]. In cases of threatened PTB, patients are likely to receive tocolytic drugs, such as nifedipine or atosiban, to attenuate uterine contractions [11]. Furthermore, patients in the study population can also receive MgSO₄, which is prescribed for either fetal neuroprotection (in case of PTB < 30 weeks’ gestation) or maternal neuroprotection (in the case of severe HDP) [11,12,27]. Patients with HDP might also be administered anti-hypertensive medications, such as labetalol, methyldopa, nifedipine, or nicardipine. 

### 2.5. Study Design 

The study comprises two periods of PPG measurements in the same study population. The primary phase will assess the effect of obstetric medications on mHRV, based on PPG data gathered throughout subjects’ hospitalization in the OCU. The secondary phase—added to compare cardiovascular features between the antenatal and postpartum periods—consists of 24 h PPG measurements at six weeks postpartum, acquired in free-living conditions at home. 

#### 2.5.1. Primary Phase

**Figure 2:**
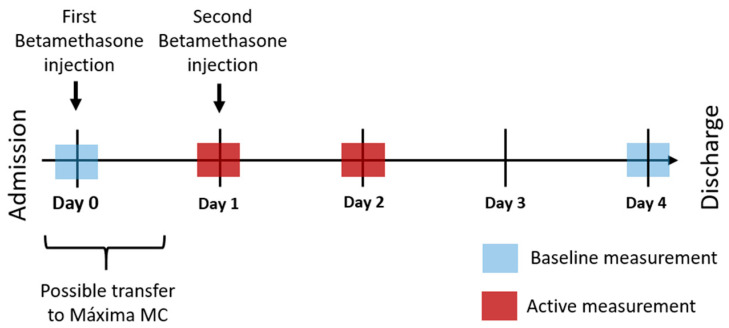
Baseline and active measurements epochs acquired in the primary phase of the study. The baseline measurements epochs are defined on day 0 and/or day 4 (light blue), while active measurements epochs are defined on day 1 and day 2 (dark red).

We specify a series of measurement epochs from our continuous measurements, as visualized in Figure 2. Our active measurement epochs (i.e., measurements to capture the effect of betamethasone) are defined on day 1 and day 2 (dark red in Figure 2). Similar studies assessing fHRV have typically found a slight increase in variability parameters on day 1, followed by a significant decrease in parameters on day 2. Hence, we will assess both active epochs against baseline measurements. We will exclude and replace subjects for whom reliable PPG data are not available in both active epochs.

We define possible baseline measurements on day 0 (i.e., before betamethasone administration) and day 4 (i.e., 72 h after the second betamethasone injection), depicted as light blue in Figure 2. Due to the typically speedy administration of betamethasone after admission, PPG measurements with the PDL will start as soon as possible to capture premedication measurements on day 0. However, many subjects will only be included on day 1 since the majority of our cohort will comprise transfers who have already received their first injection. Subsequently, we specify an additional baseline measurement on day 4 when the pharmacological effects of betamethasone will have diminished [45,46,47,48,49,50]. We will also exclude and replace subjects for whom no baseline epoch with reliable PPG data are available.

If baseline measurements from both day 0 and day 4 are available, the mean of these is taken as the baseline [35,38]. Epochs that are compared for the primary analysis will be 24 (±4) hours apart to minimize diurnal effects [38]. Selected epochs will contain at least 5 min of PPG data of quality that is sufficient to continuously determine HR [51]. Additionally, epochs will be selected from rest periods (i.e., periods without motion artefacts) where possible, since PPG are most reliable under these conditions [41,52].

#### 2.5.2. Secondary Phase

Participants will wear the PDL at six weeks postpartum for a 24-h monitoring period in free-living conditions at home. Participants are not excluded if they refuse to participate in the secondary phase. 

### 2.6. Primary and Secondary Analyses

#### 2.6.1. Primary Phase

The primary analysis will determine the effect of administering betamethasone on mHRV. Secondary analyses will, as far as possible, explore the effect of other medications on mHRV, compare cardiovascular parameters between subgroups (e.g., stratified by diagnosis), assess cardiovascular parameters during delivery, and evaluate similarities between trends in PPG and routinely acquired CTG measurements. 

#### 2.6.2. Secondary Phase

PPG measurements acquired in the secondary phase will further facilitate secondary analyses, including a within-patient comparison of cardiovascular parameters between the antenatal and postpartum periods. If the eventual sample population allows, we will also compare postpartum parameters between subgroups (stratified by diagnosis). 

### 2.7. Study Parameters

We will assess cardiovascular parameters derived from the PPG measurements to perform our analyses. These include HR, HRV features (e.g., SDNN, RMSSD, HF, LF, and pNN50) and features based on the morphology of the PPG waveform (e.g., pulse area and large artery stiffness index [53]). To describe the study cohort, we will also collect the following data from patient records:

- Maternal condition:
  o Patient characteristics, including age, BMI, and ethnicity.
  o Pregnancy characteristics, including gestational age, results of prenatal screening, and complications in pregnancy.
  o Obstetric history, including gravidity, parity, and previous pregnancy or labor complications.
  o Family history, including genetic or congenital diseases or a history of hypertension or preeclampsia.
  o Medical condition, including preexisting diseases (i.e., cardiovascular disease, pre-existing hypertension, autoimmune disorders, neurologic disorders).
  o Routine measurements, including blood pressure, laboratory test results, physical examination results, ultrasound results.
- Fetal/neonatal condition, including fetal growth, congenital diseases, birth weight, APGAR score, CTG measurements, and umbilical cord blood gases.
- Labor and delivery, including mode of delivery and clinical notes.
- Administration of medications, including timing, dosage, and reasons for administration.

The electronic medical records from the hospital only contain information relevant to a patient’s hospitalization or appointments at Máxima MC. Subsequently, we will contact subjects who did not deliver at Máxima MC to retrieve basic details of their delivery (i.e., birth weight and gestational age). 

For subjects who participate in the secondary phase and have their postpartum appointments at Máxima MC, information on their postpartum condition (e.g., postpartum complications and standard checkup measurements) will also be collected from their electronic medical records. 

### 2.8. Subject Inclusion and Exclusion Criteria

Patients admitted to the OCU at Máxima MC who are going to receive one or both dosages of betamethasone injection(s) are eligible for inclusion. Table 1 outlines the entire inclusion and exclusion criteria. 

Retrospectively, if subjects are identified to be incorrectly enrolled (i.e., not meeting the full eligibility criteria), they will be excluded from the study analysis and replaced with a new subject. 

### 2.9. Sample Size

We designed the study to detect the differences in mHRV indices between the active and baseline measurement epochs (in line with our primary analysis). Since we will assess multiple HRV indices, we base our sample size calculation on detecting a difference in mean NN intervals (i.e., the time between heartbeats). NN intervals form the basis for calculating HRV indices and are also a less sensitive measure than HRV, therefore, resulting in a conservative sample size estimation. Subsequently, we calculated the sample size using a two-sided *t*-test for a confidence interval of 95% and a power assumption of 80%. The expected variation in NN-interval measurements was estimated from studies assessing NN intervals in pregnant populations [14,54,55]. No prior research was available to guide the decision concerning effect size but given the heterogeneity of the cohort we would like to be able to detect a small effect. Subsequently, we selected an effect size of 10%. 

Based on these parameters, we calculated a sample size of 43 using the built-in power function for a paired t-test in R (version 3.5.3, RStudio Inc., Boston, MA, USA). Adding in a safety margin of 20% to the expected variation, the sample size increases to 61. We will perform an interim analysis after including 43 subjects to assess whether further inclusions are necessary. The study is powered for the primary analysis, and not specifically powered for secondary analyses. 

### 2.10. Statistical Analysis 

Our study cohort has various diagnoses, which can result in different baseline measurements for mHRV. Owing to this, a within-subject comparison of mHRV features will be performed for the primary analysis to minimize the effect of this heterogeneity. For each participant, the active measurement epochs on day 1 and day 2 will, respectively, be compared to the baseline measurement epoch. 

Since the treatment of this study cohort is guided by standard clinical management, the co-administration of medications is unavoidable [11,12,27]. To this end, we will perform variance analyses to understand whether this co-administration affects our results. For secondary analyses in both phases, we will perform within-subject as well as between-group comparisons. 

We will test normality assumptions with a Shapiro-Wilk test and subsequently compare continuous variables using a paired *t*-test or a Wilcoxon matched-pairs test, and categorical variables using a χ2-test or Fisher’s exact test, chosen as appropriate. *p* < 0.05 is considered significant for a two-tailed test. Effect sizes will be reported along with the *p* values [56].

### 2.11. Data Handling and Storage

We will adhere to the European General Data Protection Regulation (GDPR) and the Dutch Personal Data Protection Act (“Uitvoeringswet AVG”) for data processing and analyses. Subsequently, subject data will be de-identified. 

We will use Research Manager (version 5.51.0, Research Manager, Deventer, The Netherlands) for the case report form and data handling. Personal data will be stored according to Good Clinical Practice guidelines. Analyses are carried out under the Eindhoven MedTech Innovation Center framework, in collaboration between Máxima MC, Philips Research, and the Eindhoven University of Technology.

### 2.12. Ethics and Dissemination 

The Medical Ethics Committee of Máxima MC, Veldhoven, The Netherlands, confirmed that the study neither imposes any changes in general practice nor does it burden participants. Therefore, in line with the Declaration of Helsinki, a waiver for ethical approval was granted (N19.112; 02/12/2019). The study is registered in the Dutch Trial Register (NL8204; 06/12/2019).

All investigators agree to publish the study results in an international peer-reviewed journal, regardless of whether the outcomes align with the stated hypotheses. The full study protocol is available upon request. 

## 3. Discussion

The autonomic dysfunction associated with pregnancy complications has increasingly been studied by investigating mHRV [17,19]. However, the mHRV features obtained in these cohorts are possibly confounded by routinely administered obstetric medications—in particular, corticosteroids. This likely impedes the accurate interpretation of such results and could explain why they are often conflicting. Therefore, quantifying changes in mHRV in response to obstetric medications would not only enhance our understanding of how these medications affect maternal physiology, but also improve the interpretation of past and future studies. 

Our study is one of only a few to explore the effect of administering routine obstetric medications on mHRV [33,57], and the first to focus on investigating changes in mHRV resulting from the antenatal administration of corticosteroids (betamethasone). Apart from a small number of human and animal studies [32,58,59,60], research has focused on assessing changes in fHRV—demonstrating that administering betamethasone significantly decreases fHRV parameters [35,38,61]. Since fHRV is not continuously monitored in cohorts hospitalized due to pregnancy complications, these fetal studies (such as that of Verdurmen et al.) had to deliberately incorporate fHRV measurements into clinical workflow, which can be logistically challenging [38]. Since our clinical setting and protocol are comparable to theirs, we implement unobtrusive monitoring to ensure that our study fits more seamlessly into standard clinical workflow. 

For collecting mHRV in our study, we selected a wristwatch-like device (the PDL) owing to its ease of use and limited interference with clinical workflow. The traditional alternative would be an ECG Holter monitor, as it might offer higher accuracy in determining mHRV. However, this approach is more obtrusive and cumbersome for both the patient and clinical staff. Furthermore, in addition to high participant compliance in wrist-worn monitoring in pregnant populations [62], HRV determined from PPG measurements, sampled above 25 Hz (PDL: 32 Hz), can be as reliable as that calculated from ECG [63]. Epochs used for analyses will be selected from rest periods where possible since this is when PPG measures are generally most reliable. Still, since frequency domain features may be less reliable when calculated from PPG measurements, we will interpret these features with caution [52]. 

The unobtrusive nature of wrist-worn PPG measurements also offers opportunities for additional exploratory analyses: firstly, a continuous dataset representing the complete period of hospitalization of participants can be collected; secondly, it enables us to collect 24 h of postpartum at-home measurements for these same participants (i.e., the secondary phase). Incorporating all these measurements could allow for the analysis of mHRV throughout the perinatal period (i.e., antepartum, intrapartum, and postpartum), which—to our knowledge—has not yet been assessed. Insights into the postpartum period could be particularly useful, since literature on how autonomic regulation changes in this period is limited [64,65,66].

Defining a baseline measurement epoch is another important challenge in assessing the effect of betamethasone on mHRV. The presumptive ideal is the epoch leading up to the first betamethasone injection (i.e., day 0 in Figure 2), but this is impractical given that most of our study cohort will be transfers who have already received their first injection. Furthermore, since admission is typically urgent and unexpected, patients are likely physiologically stressed during day 0, which can affect HRV parameters [67]. Therefore, an alternative baseline measurement is necessary. Guided by the available literature and the pharmacokinetics of betamethasone, we define our alternative baseline measurement on day 4. Koenen et al. found that, while administering betamethasone suppresses the diurnal rhythm of maternal cortisol and ACTH levels, this rhythm returns by day 4 [32]. This aligns with what is known concerning the pharmacokinetics of the medication in the maternal system, with studies showing the maternal terminal half-life of betamethasone as 6 to 12 h [45,46,47,48], and the corresponding biological half-life as 36 to 59 h [49,50]. Several studies have also shown that the effect of betamethasone on fHRV ceases by day 4 [35,37,38]. Factoring in that Ballard et al. have demonstrated that the medication’s terminal half-life in the maternal system is half of that in the fetus [46], it is reasonable to assume that day 4 is a conservative baseline measurement. In the case that both baseline epochs are available, we use their mean [35,38].

For the results of the study to be applicable in clinical practice, participants will represent a cohort of women who typically receive corticosteroids, i.e., patients with varying characteristics and diagnoses (e.g., HDP, threatened PTB), and who subsequently receive multiple medications. The heterogeneity in characteristics and diagnoses could serve as limitations, as they will likely also influence mHRV. We account for this heterogeneity by focusing on within-patient comparisons when assessing the effect of betamethasone on mHRV, emphasizing the relative change between the active and baseline epochs, and averaging results across subjects. Hence, the effect of the heterogeneity on the study results will be reduced. 

Another limitation is the possible confounding effect of co-administration of medications. This is unavoidable in this study design and cohort [11,12,27]. As previously mentioned, little literature exists on the effect of obstetric medications on mHRV, aside from one study, which determined that a tocolytic drug had no significant effect on mHRV [33]. We aim to assess the impact of this co-administration by doing variance analyses when multiple medications have been administered. 

Still, the most prominent knowledge gap concerns betamethasone, and we subsequently focus on investigating the effect of this medication on mHRV. Results from this study could identify the possible confounding effect of betamethasone on mHRV, thereby improving the interpretation of existing and future studies assessing the autonomic dysregulation associated with pregnancy complications, such as HDP or threatened PTB. An improved interpretation of the changes in mHRV in these cohorts could facilitate earlier diagnosis through tracking deteriorations in mHRV. In turn, early detection could enable the prevention or better management of these complications, alleviating some of the burdens they place on women, families, and society. 

## Figures and Tables

**Table 1:** Inclusion and exclusion criteria for MAMA-hart study.

Inclusion Criteria	Exclusion Criteria
− Age 18 years and above − Gestational age 23 5/7 to 33 6/7 weeks − Yet to receive the second betamethasone injection − Proficient in Dutch or English	− History of severe arrhythmia and/or maternal congenital heart disease − Diseases with known effects on ANS Known allergies to hard plastic (e.g., used in sport watches) or elastic band material − Wounds, injuries, or infectious diseases on the wrist where the PDL will be worn − Tattoo location on wrist interfering with the positioning of the PDL − Both wrists unavailable for wearing the PDL (e.g., owing to intravenous lines) − Dexamethasone (another brand of corticosteroid) administered instead of betamethasone

PDL = Philips Data Logger.

## Data Availability

The data associated with this study is publicly unavailable. Upon reasonable request, in accordance with patient consent and with permission of relevant parties, the de-identified data might be made available to others by the corresponding author. Additionally, the full study protocol is available upon request.
